# Up to 52 administrations of macrocyclic ionic MR contrast agent are not associated with intracranial gadolinium deposition: Multifactorial analysis in 385 patients

**DOI:** 10.1371/journal.pone.0183916

**Published:** 2017-08-31

**Authors:** Ji Ye Lee, Ji Eun Park, Ho Sung Kim, Seon-Ok Kim, Joo Young Oh, Woo Hyun Shim, Seung Chai Jung, Choong Gon Choi, Sang Joon Kim

**Affiliations:** 1 Department of Radiology, Soonchunhyang University Bucheon Hospital, Wonmi-gu, Bucheon, Korea; 2 Department of Radiology and Research Institute of Radiology, University of Ulsan College of Medicine, Asan Medical Center, Seoul, Korea; 3 Department of Clinical Epidemiology and Biostatistics, University of Ulsan College of Medicine, Asan Medical Center, Seoul, Korea; Teikyo University School of Medicine, JAPAN

## Abstract

**Purpose:**

To determine whether multiple repeated administrations of gadolinium-based macrocyclic ionic MR contrast agent (MICA) are associated with intracranial gadolinium deposition and identify the predisposing factors for deposition in various clinical situations.

**Materials and methods:**

In this institutional review board-approved retrospective study, 385 consecutive patients who underwent MICA-enhanced MR imaging were enrolled. The dentate nucleus-to-pons (DN/P) and globus pallidus-to-thalamus (GP/Th) signal intensity (SI) ratios on unenhanced T1-weighted images were recorded by 2 independent readers and averaged. The mean DN/P and GP/Th SI ratio difference between the last and the first examinations were tested using the one-sample *t*-test. Student’s *t*-test and stepwise regression analysis were used to identify the predisposing factors for deposition based on the number of administrations, time interval, hepatic and renal function, magnetic field strength, and chemo- or radiation therapy.

**Results:**

The mean DN/P SI ratio difference was not different from zero (*P =* .697), even in patients with ≥20 administrations (n = 33). Only patients with abnormal renal function showed an increase in the mean DN/P SI ratio difference (*P* = .019). The mean DN/P SI ratio difference was not associated with any predisposing factors. However, the mean GP/Th SI ratio difference showed decrease (*P* < .001), which was associated with age (*P* = .007), number of administrations (*P* = .01) and number of radiation therapy sessions (*P* = .022) on multivariate analysis.

**Conclusion:**

Multiple repeated administrations of MICA were not associated with increased T1 signal intensity in deep brain nuclei suggestive of Gd deposition in patients with normal renal function.

## Introduction

Though a recent large cohort study showed no association between gadolinium exposure and parkinsonism [1], the *in vivo* stability of gadolinium-based contrast agents (GBCA) remains an important issue. The chemical structure of GBCA is critically important in gadolinium exposure considering that the linear chelate complex imposes a greater risk of gadolinium release compared to macrocyclic complex [2–4]. After linear GBCA use, T1 hyperintensity is shown to occur not only in the dentate nucleus (DN) and globus pallidus (GP), but also in various other brain regions [5] and a dose-dependent relationship has been shown [6–14]. Meanwhile macrocylic GBCAs are recognized as a relatively stable contrast agent, and experimental studies have not found any causal relationship for T1 high signal intensity (SI) in the DN and/or GP [7, 8].

Many clinical studies have reported a T1 SI change for the 3 commercially available macrocyclic GBCAs- gadoteridol, gadobutrol, and gadoterate meglumine, but these increases were not found to be statistically significant [15–17]. One clinical study reported the association between T1 high signal intensity in the DN and use of gadobuterol [18], but the study design included a homogeneous group of patients with a specific disease, and thus the generalizability of this study is debatable [8, 19, 20]. Various other clinical conditions—including multiple contrast agent administrations, imaging interval, or treatment regimen—may be predisposing factors for intracranial gadolinium deposition. Previous studies, in which number of patients ranged from 30 to 50, have lacked the statistical power to address this issue.. Thus, a clinical study based on these various other clinical factors that has a sufficient population size, may be needed to demonstrate an association between GBCA use and intracranial gadolinium deposition.

Since 2009, a macrocyclic ionic MR contrast agent (MICA) has been used as a primary GBCA at our hospital. We tried to explore various clinical predisposing factors for gadolinium deposition by including patients in whon only MICA was used. We hypothesized that the T1 high SI in the DN and GP may be associated with MICA use, and we tried to account for various clinical situations including multiple MICA administrations (>20 times), the time interval between examinations, hepatic or renal function, and chemo-or radiation therapy. Thus, the purpose of our study was to determine whether multiple repeated administrations of MICA are associated with intracranial gadolinium deposition and identify the predisposing factors for deposition in various clinical situations.

## Materials and methods

### Patient data

**Fig 1:**
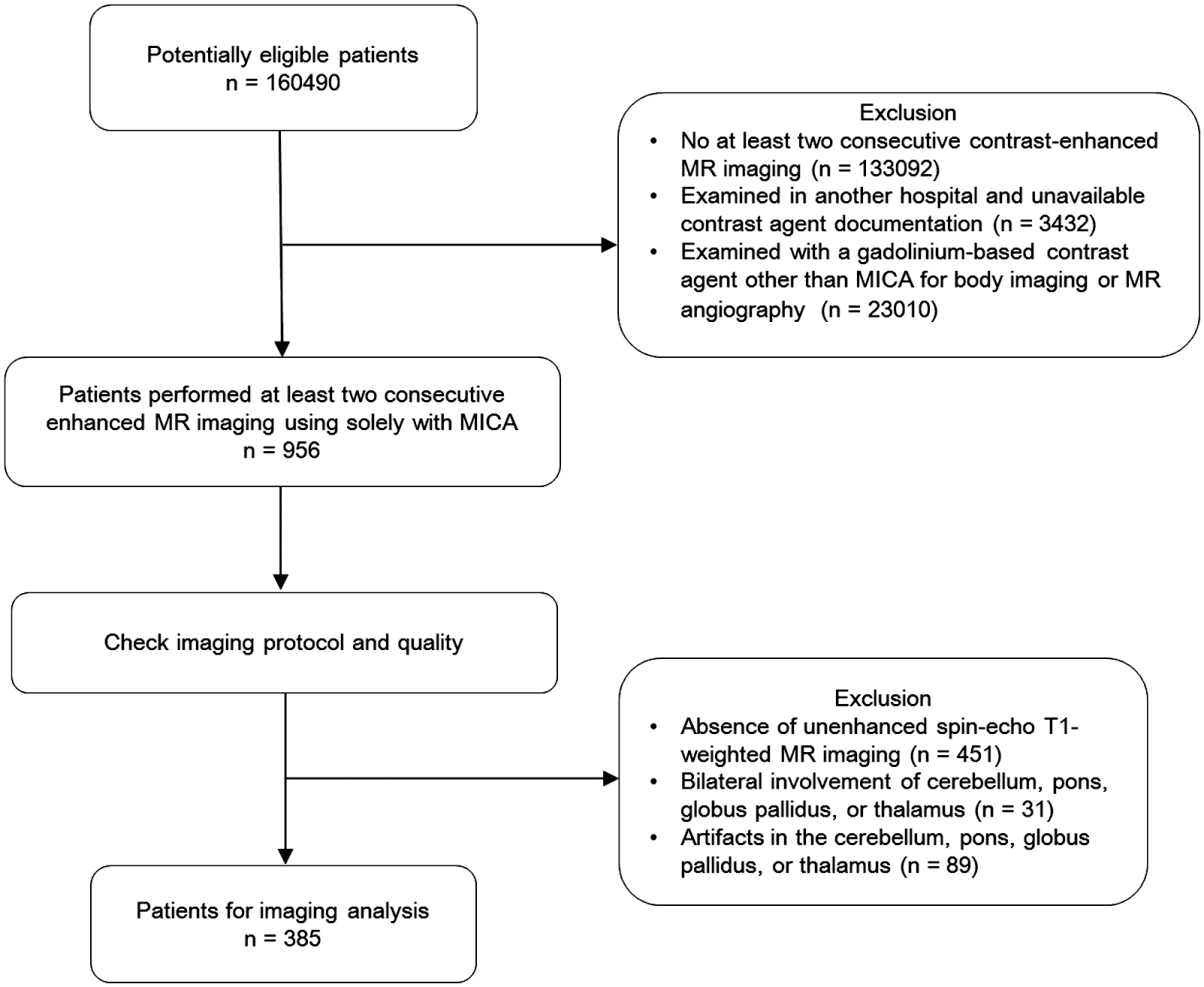
Flow diagram of patient selection. MICA, macrocyclic ionic MR contrast agent.

The protocol of this retrospective study was approved by the institutional review board of Asan Medical Cetner and the need for informed consent was waived. The picture archiving and communication systems of our institution, wherein > 30000 brain MR examinations are performed annually, was searched from March 2009 to July 2016 to identify patients who met the following inclusion criteria: (a) unenhanced T1-weighted images were obtained before the first GBCA administration; (b) at least 2 consecutive MR examinations were performed with contrast-enhanced study in the former; and (c) all of the consecutive MR imaging examinations were performed exclusively at our institution, and followed with the same field strength (1.5 Tesla or 3.0 Tesla). The exclusion criteria were as follows: (a) lack of contrast-enhanced MR examinations (n = 133092); (b) examined in another hospital using GBCA (n = 3432); and (c) examined using a GBCA other than macrocyclic ionic GBCA for body imaging or brain MR angiography according to our institution’s protocol (n = 23010). Prior to the imaging analysis, the image protocol and quality were assessed for all examinations. As the quantitative analysis of SI on T1-weighted spin-echo (SE) sequence is not interchangeable with the gradient-echo sequence [11], examinations that did not include unenhanced T1-weighted spin echo images were excluded (n = 451). Additionally, examinations with bilateral involvement of edema, tumor, or other lesions located in either the cerebellum, pons (P), GP, or thalamus (Th) (n = 31), as well as those with artifacts in the cerebellum, P, GP, or Th (n = 89) were excluded. A total of 385 patients (mean age, 56.8 years; age range, 18–90 years; 172 men and 213 women) with available data on the first and the last examinations with unenhanced T1-weighted spin-echo images were included. A summary of the patient inclusion process is shown in Fig 1.

Patients were evaluated with routine laboratory tests before the MR examinations. Patient data, including the sex and age of the patients, indication for MR imaging, renal and hepatic function, and a history of chemotherapy or radiation therapy, were obtained from electronic medical record,. Abnormal renal function was defined as an estimated glomerular filtration rate (eGFR) ≤ 60mL/min/m^2^. Severely impaired renal function was defined as an estimated glomerular filtration rate of 45 mL/min/1.73 m^2^ or acute renal failure. Abnormal hepatic function was defined as abnormal serum concentrations of aspartate aminotransferase, alanine aminotransferase, γ-glutamyl trans-peptidase, and total bilirubin. Radiation therapy was categorized as whole-brain irradiation and localized irradiation, excluding the DN or GP.

### Imaging acquisition

MR imaging was performed primarily with a 3.0-T MR scanner (Achieva or Ingenia, Philips Medical Systems, The Netherlands; or Skyra, Siemens Healthcare, Germany) or a 1.5-T MR scanner (Achieva; Philips), using an eight-channel head coil. The brain MR protocol with a spin-echo sequence included T1-weighted imaging (T1WI), T2-weighted imaging, fluid-attenuated inversion recovery imaging, and contrast-enhanced T1WI. The range of axial unenhanced T1-weighted fast spin echo images were obtained using the following parameters: repetition time (TR)/echo time (TE), 450–550 msec/7.3–11 msec; section thickness, 5–7 mm; field of view (FOV), 20–25 cm; and matrix, 200 × 256.

Contrast enhancement was achieved using gadoterate meglumine (Dotarem, Guerbet, Paris, France). A standardized single dose of 13 ml of MICA was exclusively applied in most adult patients for contrast-enhanced MR examination. For perfusion MR including dynamic susceptibility contrast imaging or dynamic-contrast enhanced imaging, a double injection of MICA was applied. The number of MICA administrations and the interval between the first and the last examinations were recorded. The mean interval between GBCA administrations was calculated by dividing the time (in days) between the first and last MRI examinations by the total number of examinations minus 1.

### Image analysis

**Fig 2:**
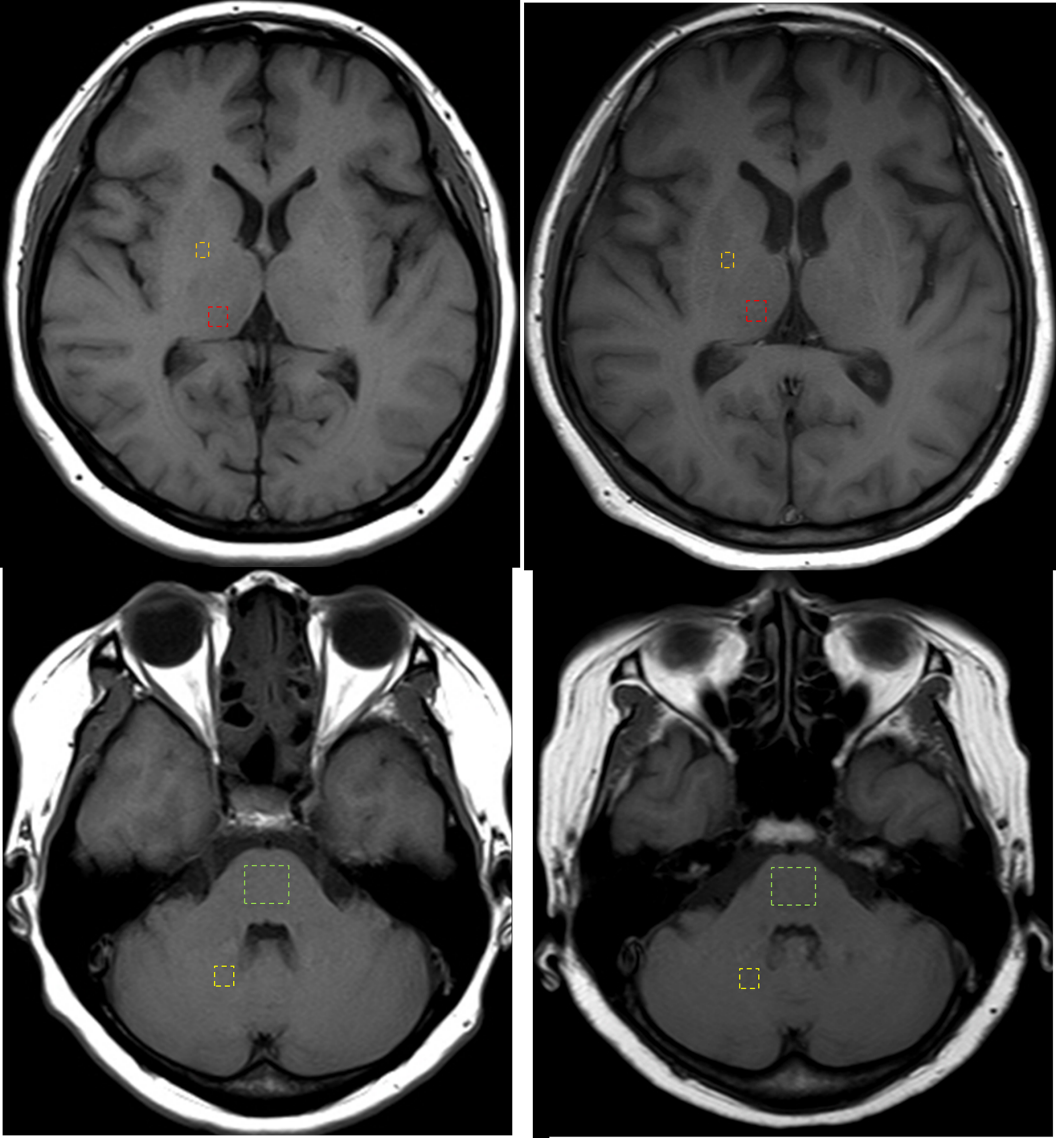
Axial T1-weighted MR images at the level of the basal ganglia and dentate nucleus. The regions of interest used in the quantification of signal intensity are shown as dashed lines for the globus pallidus (GP, orange), thalamus (Th, red), dentate nucleus (DN, yellow), and pons (P, green). (upper row) GP and Th in the first examination and after 21 administrations of macrocyclic ionic MR contrast agent. (lower row) DN and P in the first examination and after 21 administrations of macrocyclic ionic MR contrast agent.

Two radiologists (J.E.P. and J.Y.L., with 4 and 2 years of experience in neuroradiology, respectively), blinded to the clinical data and number of exams per patient, independently measured the SI in the anatomic structures by drawing regions-of-interest (ROIs) on the unenhanced T1-weighted images of each patient. Quantitative analysis of the relative T1 SI was performed as previously described [9] by placing square ROIs (minimum, 100 -mm^2^) on the right dentate nucleus (DN), central pons (P), right globus pallidus (GP), and right thalamus (Th) (Fig 2).

Before analysis, unenhanced T1- and T2-weighted images were captured in a folder. Exams from a given patient were read together, and the order of the first and last exam was not randomized. The readers were blind to the number of exams per patient, and the order of ROI measurement was randomly arranged for each patient (i.e. GP ➔ DN ➔ Th ➔ P in patient 1 and DN ➔ Th ➔ P ➔ GP in patient 2) to avoid any possible bias in the signal intensity measurement. ROIs were placed on the left side if artifacts or structural distortion due to pathology was present on the right side, and were separately recorded in these cases. When drawing the ROIs, T2-weighted images were used to guide ROI placement.

The SIs of the DN, P, GP, and Th from the 2 readers were first compared to calculate inter-reader agreement. Th averaged values of DN, P, GP, and Th from the 2 readers were then used to calculate the DN/P and GP/Th SI ratios; the mean DN/P SI ratios were estimated by dividing the mean SI of the DN by that of the P, and the mean GP-to-Th SI ratios by dividing the mean SI of the GP by that of the Th. Thereafter, the mean DN/P or GP/Th SI ratio difference was calculated between the first and the last examinations.

### Statistical analysis

All continuous variables were initially assessed for normality using the Kolmogorov-Smirnov test. Interobserver agreement between the 2 readers was assessed using the intraclass correlation coefficient (ICC) with 95% confidence intervals. The strength of agreement was classified according to common criteria as excellent (ICC > 0.75), fair to good (ICC = 0.40–0.75), and poor (ICC ≤ 0.40) [21].

A one-sample *t* test was used to determine whether the mean SI ratio difference between the first and last examinations differed from 0. An independent-sample *t* test was used to test whether the differences between 2 subgroups were significant: the mean SI ratio differences were compared between 1) patients with < 6 and ≥ 6 MR examinations; 2) patients with < 20 and ≥ 20 MR examinations; 3) patients with a mean interval < 90 days or ≥ 90 days; 4) patients with abnormal and normal renal function; 5) patients with abnormal and normal hepatic function; 6) patients who received whole-brain radiation and those who underwent tumor-selective radiation or no radiation therapy; 7) magnetic field strength; and 8) location of ROIs.

A stepwise regression analysis was performed to evaluate the predisposing factors associated with the differences in mean DN/P and GP/Th SI ratios between the first and the last examinations. The predisposing factors included age, sex, number of MICA administrations, mean interval between examinations, renal and hepatic function, type of brain radiation therapy and chemotherapy, number of radiation therapy sessions, number of chemotherapy sessions, and magnetic field strength. A *P* value of < 0.05 was considered statistically significant. Statistical planning and analysis were performed using SAS 9.3 software (SAS Institute; Cary, NC).

## Results

The patient characteristics are summarized in Table 1.

**Table 1:** Clinical characteristics of the study patients.

Characteristic	N = 385
Current age (years)	56.8 ± 13.1 (18–90)
Male-to-female ratio	172: 213
Number of MICA administrations (median, interquartile range)	5 (3–10)
Mean time interval between the first and the last examinations (days)	196.7 ± 290.4 (1.3–2441)
Indication for MR imaging	
High-grade glioma	15
Low-grade glioma	22
Other brain tumors	106
Tumors at any location in the body except the brain	201
Nonneoplastic lesion	41
Abnormal renal function	28
Abnormal hepatic function	28
History of radiation therapy	
Whole brain	34
Tumor-selective	136
History of chemotherapy	
Targeted therapy	48
Alkylating agent	196
Other chemotherapy	1
Magnetic field strength	
3.0-Tesla	331
1.5-Tesla	54

Data are presented as numbers of patients, unless otherwise specified.

MICA, macrocyclic ionic magnetic resonance contrast agent

**Fig 3:**
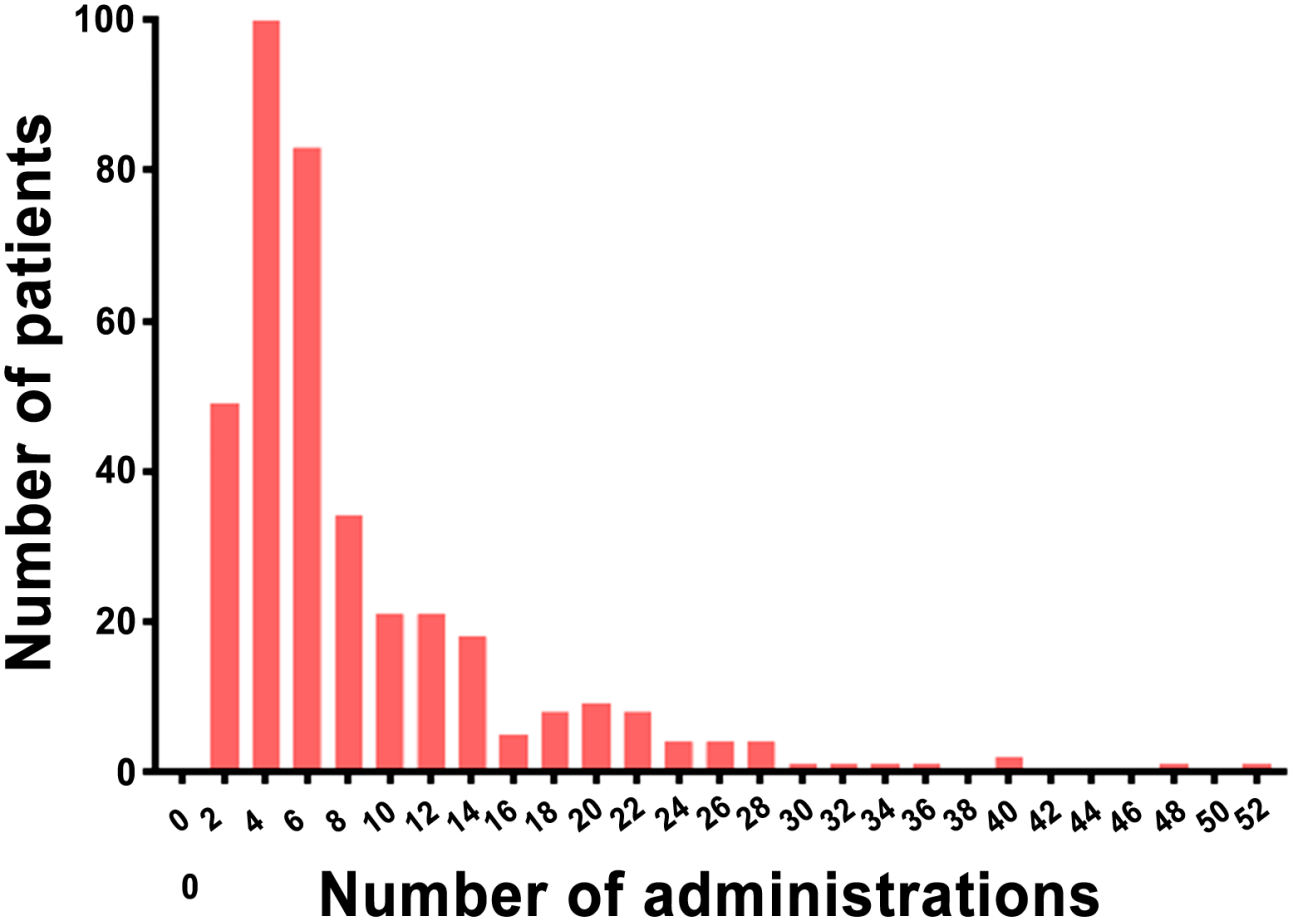
Histogram of the number of macrocyclic ionic MR contrast agent administrations in the study population.

The number of MICA administrations is shown in Fig 3, and ranged from 2 to 52 (median, 5; interquartile range, 3–10).

None of the subjects had severely impaired renal function (estimated glomerular filtration rate, 45 mL/min/1.73 m^2^) or acute renal failure. Most of our patients were indicated to undergo evaluation for metastasis (n = 201, 52.2%). Accordingly, 143 patients were found to have brain tumor(s), meanwhile 201 were found to have tumors in parts of the body other than the brain. Thirty-four patients (8.8%) underwent whole-brain radiation therapy, whereas 136 patients (28%) underwent tumor-selective radiation therapy, excluding the DN or the GP from the field. No patient received brain radiation therapy prior to the first unenhanced MR imaging. A total of 245 patients had a history of chemotherapy (63.6%).

The inter-observer agreement for quantitative analysis of SI was almost perfect for all 4 structures (range, 0.98–0.99; 95% confidence interval [CI], 0.98–1.00). The intra-class coefficient for the first examination was 0.99 (95% CI, 0.99–1.00) for DN, P, GP, and Th, whereas that for the last examination was 0.98 (95% CI, 0.98–0.99).

### Effect of MICA on SI

**Fig 4:**
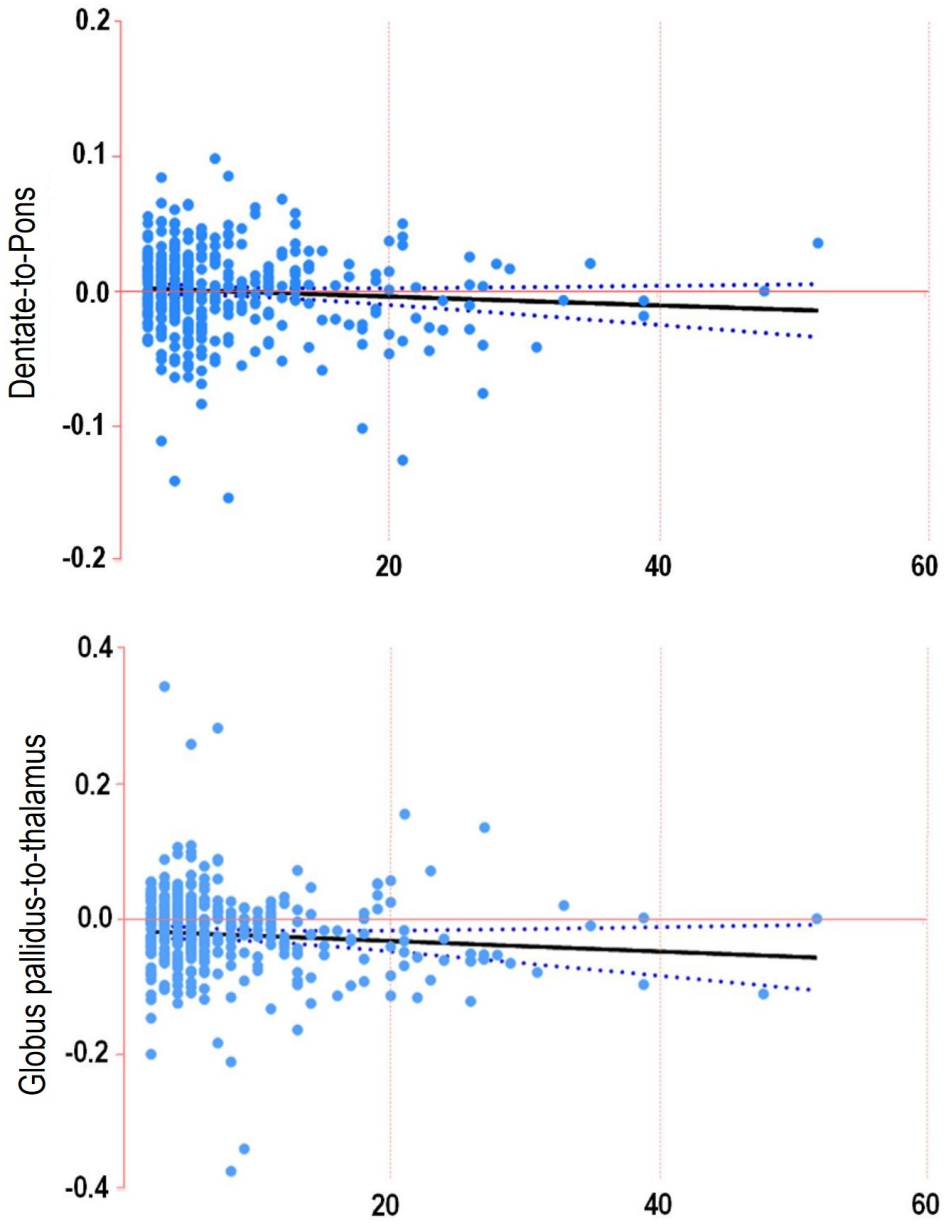
(A) Scatterplot of the mean dentate nucleus-to-pons signal intensity ratio difference with the number of macrocyclic ionic MR contrast agent (MICA) administrations. (B) Scatterplot of mean globus pallidus-to-thalamus signal intensity ratio difference with the number of MICA administrations. In each plot, the solid line represents linear regression and the dashed lines indicate 95% confidence intervals.

The mean DN/P SI ratio was 1.021 ± 0.05 (mean ± standard deviation) at the first examination and 1.021 ± 0.06 at the last examination, the difference of which did not differ from 0 (*P* = .697). The mean GP/Th SI ratio was 1.029 ± 0.08 at the first examination and 1.006 ± 0.06 at the last examination, the difference of which differed from 0 (*P* < .001); in particular, a significant decrease was noted in the last examination (difference in the mean GP/Th SI ratio, -0.021 ± 0.083). Scatterplots of the difference in the mean DN/P and GP/Th SI ratio according to the number of MICA administrations are shown in Fig 4.

Table 2 shows results of a subgroup analysis according to the number of administrations, mean time interval between examinations, renal or hepatic function, chemo- or radiation therapy, magnetic field strength, and side of ROI placement.

**Table 2:** Comparison of the mean dentate-to-pons (DN/P) and globus pallidus-to-thalamus (GP/Th) SI ratio difference according to each subgroup.

Parameters	Mean DN/P SI ratio					Mean GP/Th SI ratio				
	First	Last	SI ratio difference	P value*	P value†	First	Last	SI ratio difference	P value	P value†
All patients (n = 385)	1.02 ± 0.05	1.02 ± 0.05	0.001 ± 0.063	.697		1.03 ± 0.07	1.01 ± 0.05	-0.021 ± 0.083	< .001	
Number of administrations										
< 6 times (n = 210)	1.02 ± 0.05	1.02 ± 0.05	0.003 ± 0.058	.396	.238	1.03 ± 0.07	1.01 ± 0.06	-0.012 ± 0.060	< .001	.019
≥ 6 times (n = 175)	1.02 ± 0.06	1.02 ± 0.06	-0.004 ± 0.070	.403		1.03 ± 0.09	1.00 ± 0.06	-0.026 ± 0.077	< .001	
< 20 times (n = 352)	1.02 ± 0.05	1.02 ± 0.06	0.001 ± 0.063	.7	.15	1.03 ± 0.06	1.01 ± 0.05	-0.022 ± 0.080	< .001	.01
≥ 20 times (n = 33)	1.01 ± 0.04	0.99 ± 0.06	-0.016 ± 0.074	.236		1.02 ± 0.05	0.99 ± 0.05	-0.036 ± 0.066	.002	
Mean time interval between examinations										
< 90 days (n = 173)	1.02 ± 0.04	1.01 ± 0.06	-0.005 ± 0.066	.311	.17	1.02 ± 0.05	1.01 ± 0.06	-0.014 ± 0.072	< .001	.43
≥ 90 days (n = 212)	1.02 ± 0.05	1.03 ± 0.04	0.004 ± 0.062	.365		1.04 ± 0.07	1.01 ± 0.04	-0.031 ± 0.084	< .001	
Renal function										
Normal renal function (n = 357)	1.02 ± 0.04	1.02 ± 0.05	-0.002 ± 0.065	.649	.019	1.03 ± 0.07	1.01 ± 0.06	-0.024 ± 0.079	< .001	.798
Abnormal renal function (n = 28)	1.01 ± 0.04	1.03 ± 0.05	0.018 ± 0.038	.019		1.02 ± 0.07	1.03 ± 0.04	-0.021 ± 0.077	.165	
Hepatic function										
Normal hepatic function (n = 357)	1.02 ± 0.05	1.02 ± 0.05	0.001 ± 0.066	.972	.626	1.03 ± 0.07	1.01 ± 0.05	-0.023 ± 0.081	< .001	.587
Abnormal hepatic function (n = 28)	1.01 ± 0.04	1.00 ± 0.04	-0.004 ± 0.036	.598		1.04 ± 0.06	1.01 ± 0.04	-0.032 ± 0.059	.011	
Radiation therapy										
No radiation or tumor-selective (n = 331)	1.02 ± 0.05	1.02 ± 0.05	-0.001 ± 0.063	.907	.847	1.03 ± 0.07	1.01 ± 0.06	-0.023 ± 0.080	< .001	.735
Whole brain radiation therapy (n = 54)	1.01 ± 0.05	1.02 ± 0.08	0.001 ± 0.072	.886		1.03 ± 0.07	1.00 ± 0.06	-0.026 ± 0.071	.007	
Chemotherapy										
No history of chemotherapy (n = 138)	1.02 ± 0.05	1.01 ± 0.05	-0.007 ± 0.065	.234	.14	1.02 ± 0.06	1.01 ± 0.05	-0.014 ± 0.081	.026	.02
History of chemotherapy (n = 247)	1.02 ± 0.05	1.02 ± 0.05	0.003 ± 0.063	.391		1.03 ± 0.06	1.00 ± 0.05	-0.029 ± 0.077	< .001	
Magnetic field strength										
3.0-Tesla (n = 331)	1.01 ± 0.05	1.02 ± 0.05	0.006 ± 0.054	.053	.57	1.03 ± 0.05	1.01 ± 0.05	-0.02 ± 0.06	< .0001	.09
1.5-T (n = 54)	1.02 ± 0.03	1.01 ± 0.03	-0.003 ± 0.049	.75		1.04 ± 0.03	1.04 ±0.05	-0.004 ±0.059	.727	
Location of ROI										
Right side (n = 301)	1.02 ± 0.04	1.02 ± 0.05	-0.001 ± 0.064	.825	.70	1.03 ± 0.07	1.01 ± 0.05	-0.026 ± 0.086	< .001	.18
Left side (n = 84)	1.01 ± 0.05	1.01 ± 0.04	0.002 ± 0.064	.484		1.02 ± 0.04	1.01 ± 0.04	-0.015 ± 0.049	.008	

Data are expressed as mean ± standard deviation for continuous variables.

* are results for one-sample *t*-test

The mean DN/P SI ratio difference did not differ from 0; this trend was maintained regardless of the number of administrations (cutoff for the number of MR examinations, 6 or 20) (S1A Fig), mean time interval (cutoff, 90 days), hepatic function, history of whole-brain radiation or chemotherapy, and location of the ROI. Moreover, there was no significant difference between each subgroup using an independent-sample *t* test. Only patients with abnormal renal function showed a significantly increase in the mean DN/P SI ratio difference (0.018 ± 0.038, *P* = .019), which significantly differed from that of patients with normal renal function (*P* = .019) (S1B Fig).

The mean GP/Th SI ratio difference showed a significant decrease between the first and the last examinations (*P* < .001); this trend was maintained regardless of the number of administrations (cutoff: 6, *P* < .001; cutoff: 20, *P* < .001 or .002), mean time interval (cutoff: 90 days, *P* < .001), hepatic function (*P* < .001 or .011), history of whole brain radiation (*P* < .001 or .007) or chemotherapy (*P* < .001 or .026), and location of ROI (*P* < .001 or .008). In the patients with abnormal renal function, the mean GP/Th SI ratio difference did not decrease. Using an independent-sample *t* test, a significant difference was found according to the MR examination cutoff of 6 or 20 (*P =* .019 and .01, respectively) and a history of chemotherapy (*P =* .02), wherein a greater decrease in the mean GP/Th SI ratio difference was noted among patients with ≥ 6 MR examinations, with ≥ 20 MR examinations, and a history of chemotherapy.

### Predisposing factors for T1 high SI changes in the DN or GP in various clinical situations

The results of the stepwise regression analysis are reported in Table 3.

**Table 3:** Possible predisposing factors for the mean dentate-to-pons (DN/P) and globus pallidus-to-thalamus (GP/Th) SI ratio difference in various clinical situations.

Factors	Univariate analysis			Multivariate analysis		
	Regression coefficient	SE	*P* value	Regression coefficient	SE	*P* value
Mean DN/P SI ratio difference						
Age	0.021 (-0.028, 0.07)	0.025	.4			
Female sex	0.193 (-1.099, 1.486)	0.657	.769			
Number of administrations	-0.065 (-0.153, 0.023)	0.045	.146			
≥ 6 times	-0.788 (-2.076, 0.501)	0.655	.230			
Mean time interval	0.001 (-0.001, 0.003)	0.001	.352			
Abnormal renal function	1.960 (-0.508, 4.427)	1.255	.119			
Abnormal hepatic function	0.306 (-1.250, 1.862)	0.792	.699			
Whole-brain radiation therapy	0.181 (-1.669, 2.032)	0.941	.847			
Number of radiation therapy	-0.035 (-0.161, 0.090)	0.064	.580			
Number of chemotherapy	-0.016 (-0.088, 0.056)	0.036	.660			
Platinum-based chemotherapy	0.412 (-0.873, 1.697)	0.654	.528			
Obtained on 3.0T MR unit	0.992 (-1.070, 2.013)	1.012	.056			
Mean GP/Th SI ratio difference						
Age	-0.063(-0.123, -0.003)	0.031	.039	-0.084 (-0.144, -0.023)	0.031	.007
Female sex	0.594 (-1.002, 2.190)	0.812	.465			
Number of administrations	-0.076 (-0.185, 0.032)	0.055	.166			
≥ 6 times	-1.747(-3.332, -0.162)	0.806	.031	-2.197 (-3.872, -0.523)	0.851	.010
Mean time interval	-0.003 (-0.005, 0.000)	0.001	.053			
Abnormal renal function	0.304 (-2.753, 3.361)	1.555	.845			
Abnormal hepatic function	1.135 (-0.783,3.054)	0.976	.245			
Whole-brain radiation therapy	-0.243 (-2.529, 2.043)	1.163	.835			
Number of radiation therapy	-0.183 (-0.337, -0.028)	0.079	.021	-0.187 (-0.346, -0.028)	0.081	.022
Number of chemotherapy	-0.094 (-0.182, -0.005)	0.045	.037			
Platinum-based chemotherapy	-1.121 (-2.492, 0.251)	0.697	.109			
Obtained on 3.0T MR unit	-1.610 (-3.801, 0.582)	1.114	.149			

Note: Data in () are 95% confidence intervals.

Univariate and multivariate analyses did not show any correlation between the mean DN/P SI ratio difference and any of the predisposing factors, including patient age, sex, number of administrations, number of MR examinations ≥6, mean time interval, abnormal renal or hepatic function, types and number of radiation or chemotherapy sessions, and magnetic field strength.

In contrast, the mean GP/Th SI ratio showed a significantly negative correlation with patient age (*P* = .039; regression coefficient, -0.063; 95% CI, -0.123 to -0.003), number of MR examinations ≥6 (*P* = .031; regression coefficient, -1.747; 95% CI, -3.332 to -0.162), number of radiation therapy sessions (*P* = .021; regression coefficient, -0.183; 95% CI, -0.337 to -0.028), and number of chemotherapy sessions (*P* = .037; regression coefficient, -0.094; 95% CI, -0.182 to -0.005). Multivariate analysis indicated that patient age (*P* = .007; regression coefficient, -0.084; 95% CI, -0.144 to -0.023) (S1C Fig), number of MR examinations ≥6 (*P* = .010; regression coefficient, -0.219; 95% CI, -3.872 to -0.523), and number of radiation therapy sessions (*P* = .022; regression coefficient, -0.187; 95% CI, -0.346 to -0.028) (S1D Fig) were significant factors for a decrease in the mean GP/Th SI ratio difference between the first and the last examinations.

## Discussion

In the present study, we found that multiple repeated administrations of MICA up to 52 times are not associated with T1 high SI changes in the DN or GP. None of the predisposing factors were associated with T1 high SI changes in various clinical situations in patients with normal renal function. Interestingly, the GP/Th SI ratio decreased in the last examination, and was significantly correlated with patient age, number of administrations > 6, and number of radiation therapy sessions. As the dentate nucleus is the location where most gadolinium accumulation occurs, the lack of any observable T1 signal changes in the DN may support the current clinical use and recommendation of a macrocyclic GBCA in guidelines with safety concerns [22].

Our study results are consistent with those of the previous clinical studies of Kanda et al. [15] and Radbruch et al. [16, 17], wherein no T1 signal increase was observed in the DN after exposures to gadoteridol or gadoterate meglumine, compared to that noted after exposure to linear GBCAs. However, a recent review article indicated that all GBCAs should be evaluated individually [23] to observe effect of the agent used in terms of stability. Our study included the largest clinical data set (385 patients) with exclusive use of MICA, but we did not observe any conceivable changes in T1 SI in the DN, even in the 33 patients who underwent > 20 administrations. This stability of MICA is distinguished from that of linear GBCA, which exhibited extensive intracranial deposition even in the substantia nigra, posterior thalamus, and putamen in cases with > administration in a recent study [5].

Multiple conditions other than known predisposing factors including multiple sclerosis, Fahr disease, hypoparathyroidism, neurofibromatosis, and inherited metabolic disorders [23, 24] may cause T1 high SI changes. However, it is not possible to control for all potential conditions in clinical settings when assessing a small population. Therefore, the enrollment of a sufficient number of patients may facilitate the exploratory analysis of potential predisposing factors that remain unknown. We attempted to evaluate the influencing factors for intracranial gadolinium deposition, such as the number of administrations, mean time interval between examinations, hepatic or renal function, and history of radiation or chemotherapy, by including at least 28 patients in each group. Moreover, the stepwise regression analysis enabled the entry and deletion of each variable to explore their possible association with T1 signal changes, and found no predisposing factors associated with the DN/P SI ratio in patients with normal renal function.

Abnormal renal function was the only predisposing factor for an increase in DN/P SI ratio; this relationship may be explained by the fact that all of the MICA may be excreted via the kidney [3], and the delayed excretion enables the chelates to release gadolinium ion. Another hypothesis is that increased endogenous anions (i.e. phosphate) due to impaired renal function may compete with gadolinium ion and leads to its dissolution from the chelates [23]. Although the mechanism and clinical significance of intracranial gadolinium deposition are unclear, our findings support the careful use of MICA in patients with impaired renal function. Also, these findings may be related to gadolinium deposition in the body, which is itself a predisposing factor for nephrogenic systemic fibrosis [23, 25].

Interestingly, the GP/Th SI ratio decreased in the last examination; this decrease was significantly associated with time-related factors, including age, number of administrations >6, and the number of radiation therapy sessions. A similar negative relationship for the use of MICA was observed for the dentate-to cerebellar cortex ratio in rat brain [12]; the authors proposed that the decrease in SI in the cerebellar cortex may have been caused by the use of hyperosmolar saline in the experimental setting or may have been the effect of normal aging. Our results support the latter hypothesis, as isolated gadolinium deposition in posterior thalamus is not likely, and the decrease in the GP/Th SI ratio was associated with aging-related factors on multivariate analysis. This suggests that age can be a confounding variable in the quantitative measurement of T1 high SI in the GP or posterior Th, and future studies need to consider this effect when placing ROIs in these areas.

The present study had certain limitations. First, the retrospective design of our study limits our ability to identify the causal relationship between MICA administrations and T1 signal changes, particularly for the GP/Th SI ratio. Second, there may be interactions between concurrent systemic treatments and T1 signal changes. However, we attempted to identify the variables that can potentially cause T1 hyperintensity by performing subgroup and stepwise regression analyses in a relatively large number of patients. Third, though our study included only spin-echo T1-weighted images, the range of imaging parameters of different TR/TE combinations may have caused differences in signal intensity. We tried to control for this feature by calculating SI ratio differences with the normalization method. Also, we performed a subgroup analysis for different magnetic field and found that T1 high signal change was not affected by field strength. Fourth, the selection of only 4 sites in the brain—including the DN, GP, P, and Th—in the present study has inherent limitations, as it is impossible to completely rule out intracranial gadolinium deposition in other sites. However, there are no alternatives since tge dentate nucleus and globus pallidus are recognized as the sites with the highest *in vivo* accumulation of gadolinium. Finally, we acknowledge that MRI has limited sensitivity for evaluating gadolinium deposition in brain tissues since recent histopathologic human studies have shown gadolinium deposits present in all evaluated brain tissues after the administration of macrocyclic agent [26].

In conclusion, multiple repeated administrations of macrocyclic ionic MR contrast agent in various clinical situations are not associated with intracranial gadolinium deposition in patients with normal renal function. Caution is warranted in patients with abnormal renal function. Our research supports the current extensive use of macrocyclic GBCAs, particularly the use of macrocyclic ionic agents. Future studies using large clinical data to compare different macrocyclic GBCAs may be helpful to assess their *in vivo* stability and safety.

## Supporting information

S1 Fig (A) Example of multiple GBCA administrations, (B) example of a patient with abnormal renal function, (C) example of a patient with aging, and (D) example of a patient with history of whole brain radiation therapy.
(DOCX)
