# Supplementary material for: Up to 52 administrations of macrocyclic ionic MR contrast agent are not associated with intracranial gadolinium deposition: Multifactorial analysis in 385 patients
Source: PLoS One. 2017 Aug 31;12(8):e0183916. doi: 10.1371/journal.pone.0183916 (PMC5578663; doi:10.1371/journal.pone.0183916)

**S1A Fig.** Axial T1-weighted MR images on 3.0-Tesla (field of view, 230 x 230 mm) of a patient before (left) and after 20 (middle) after 33 (right) administrations of the macrocyclic ionic GBCAs. No definite T1 signal increase is noted in the dentate nucleus.


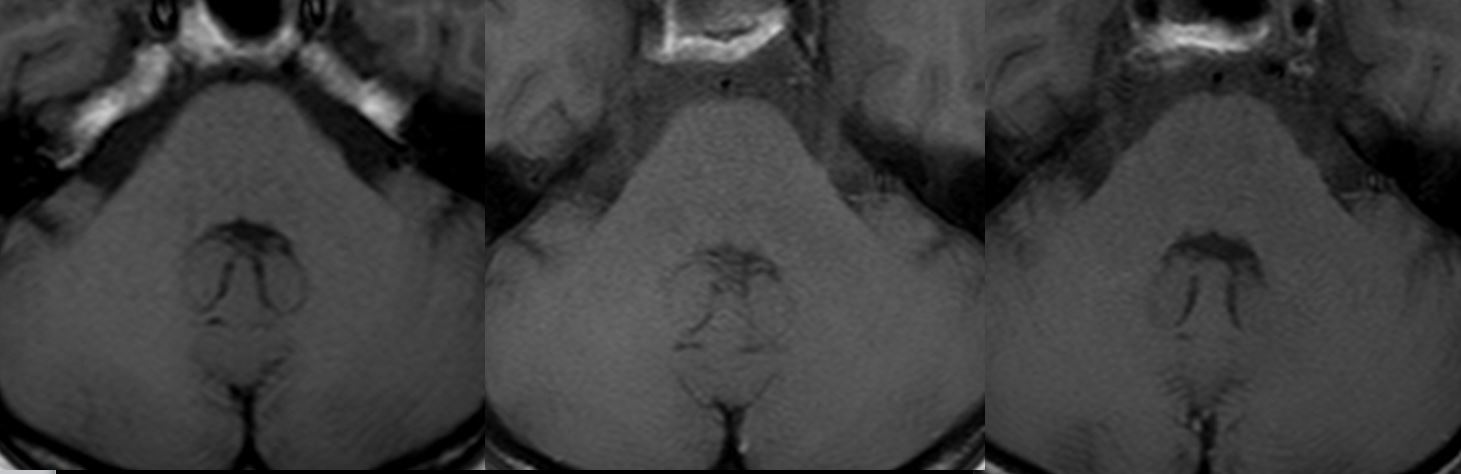


**S1B Fig.** Axial T1-weighted MR images on 3.0-Tesla (field of view, 230 x 230 mm) of a patient with abnormal renal function before (left) and after 13 (right) administrations of the macrocyclic ionic GBCAs. Note that increase of T1 signal intensity in the dentate nucleus.


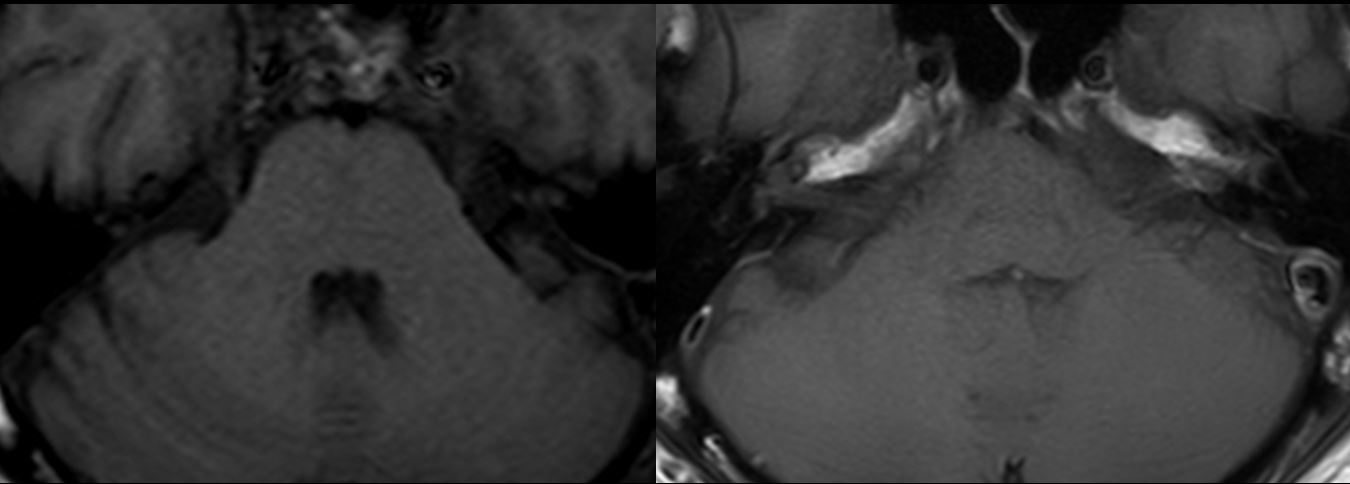


**S1C Fig.** Axial T1-weighted MR images on 3.0-Tesla (field of view, 230 x 230 mm) of a patient at age of 41 (left) and at age of 61 (right), with 7 administrations of the macrocyclic ionic GBCAs. Note that decrease of T1 signal intensity in the globus pallidus.


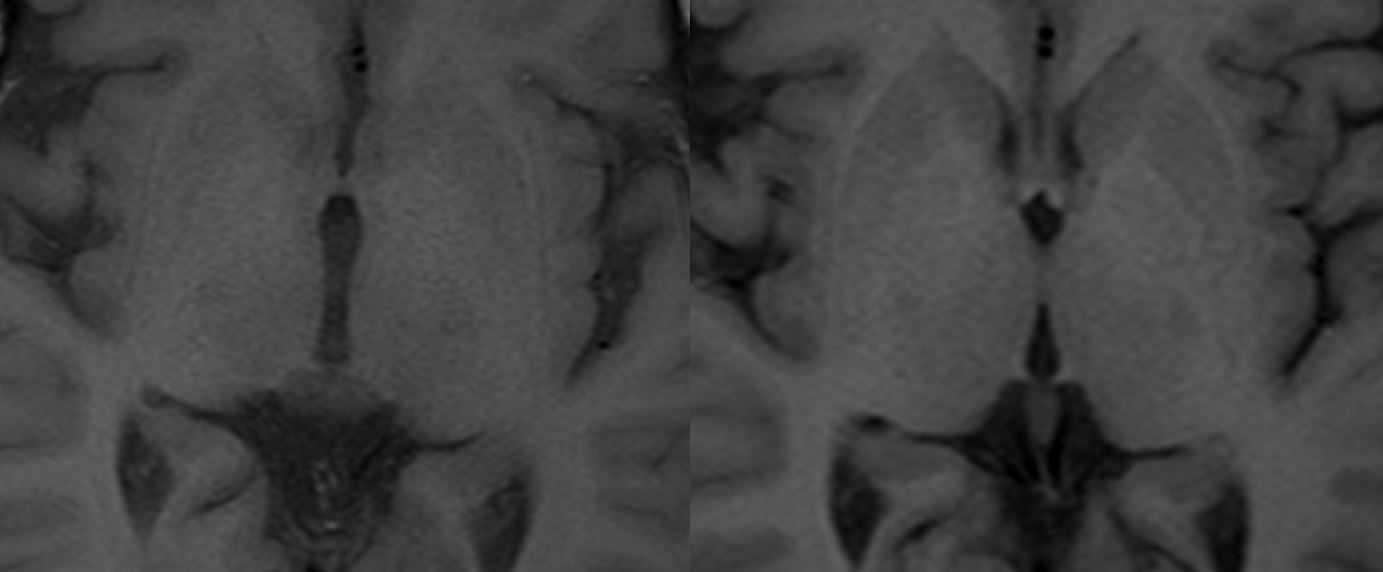


**S1D Fig.** Axial T1-weighted MR images on 1.5-Tesla (field of view, 210 x 210 mm) of a lymphoma patient received whole brain radiation therapy. MR images were obtained before (left) and after (right) radiation therapy and 21 administrations of the macrocyclic ionic GBCAs. Note that decrease of T1 signal intensity in the globus pallidus.


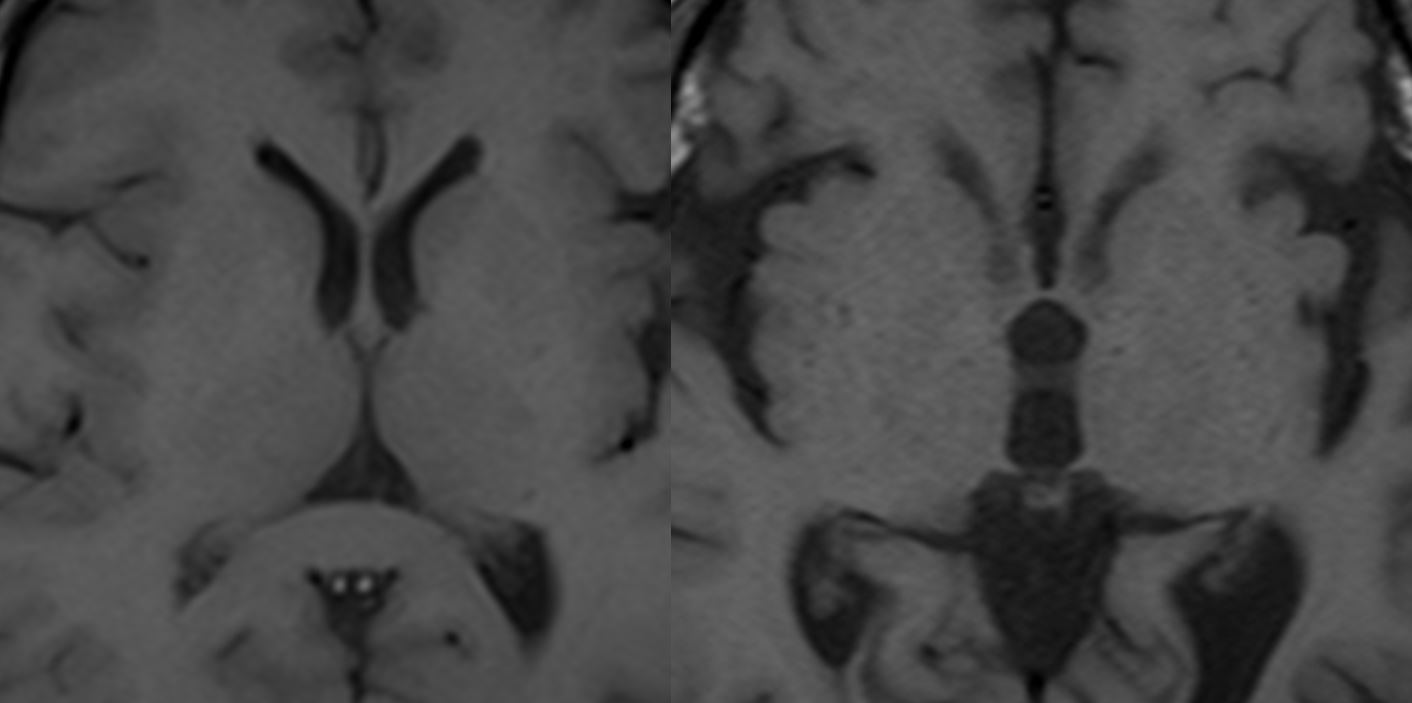

Supplement: S1 Fig — (A) Example of multiple GBCA administrations, (B) example of a patient with abnormal renal function, (C) example of a patient with aging, and (D) example of a patient with history of whole brain radiation therapy. (DOCX) [file pone.0183916.s001.docx]
